# Pricing and assembly rate decisions for a prefabricated construction supply chain under subsidy policies

**DOI:** 10.1371/journal.pone.0261896

**Published:** 2022-01-06

**Authors:** Wen Jiang, Xian Qi

**Affiliations:** College of Architecture and Urban-Rural Planning, Sichuan Agricultural University, Chengdu, P.R. China; Sunway University, MALAYSIA

## Abstract

Prefabricated construction has attracted worldwide concern and promotion due to its environmental friendliness, high quality, and high efficiency. In China, the application of prefabricated construction still lags due to its high cost. To improve prefabricated construction development, the Chinese government and provinces have launched subsidy policies for different objects that offer subsidies to the assembler, the manufacturer, or consumers. Subsidy policies for different subsidy objects have different impacts on the manufacturer wholesale price and assembler retail price and assembly rate and make their decisions more complicated. Therefore, this study uses game theory and builds three models to analyze the effects of government subsidies on manufacturer pricing, assembler pricing, assembly rate decisions, and profit. We find that government subsidy policies can bring more profit to prefabricated construction enterprises, reduce their costs, and benefit the promotion of prefabricated construction. Through comparison and numerical analysis, we also find that when the government subsidizes enterprises more, it is better to subsidize the assembler, because it is good for all three parties. First, consumers can obtain a lower retail price. Second, enterprises can obtain more profits. Finally, for the government, this approach can increase the demand for prefabricated construction and increase the assembly rate, which is conducive to the promotion of prefabricated construction. When the government subsidizes customers more, it is better for the assembler and the manufacturer to subsidize customers, because they can obtain more profits. It is better for the government and customers to subsidize the assembler or the manufacture, because consumers can get the lower retail price. Although the assembly rate and enterprises’ profits are not optimal, they have also been improved. In addition, when the government directly subsidizes enterprises, the enterprises will actively cooperate with the subsidy policy and are more willing to adopt prefabricated construction. This approach will benefit the promotion of prefabricated construction.

## Introduction

In recent years, global warming has become an increasingly serious issue. Many studies have shown that a large amount of carbon dioxide and other greenhouse gas emissions are the main causes of global warming [1, 2]. The construction industry is the largest single emitter of CO_2_ and an important consumer of energy [3]. The construction industry contributes up to 40% of the total energy consumption and 36% of the CO_2_ in the European Union [4]. Traditional construction technology contributes serious pollution to the surrounding environment and is no longer able to adapt to the low carbon development model of modern society [5]. As a good alternative to the conventional method [6], prefabricated construction has the basic characteristics of standardization, prefabrication in factories, and scientific management [7]. Moreover, it plays a significant role in cleaner production in the construction industry and has the advantages of reducing construction waste, improving quality control, saving time, reducing labor demand, and reducing resource consumption [8–10]. With the government and the public increasing demand for environmental protection, various countries are vigorously promoting prefabricated construction. For example, the promotion of prefabricated construction has been a strong focus of the Chinese government [11]. Prefabricated construction is also a key driver for innovation in the New Zealand house-building industry, and the government is actively promoting its development. Government initiatives, such as KiwiBuild, that aim to increase the supply of housing to meet the demand are reliant on prefabricated construction [12]. Australia is also actively developing prefabricated construction, and its market for prefabricated construction is growing [13]. In Japan, prefabricated construction has dominated a significant proportion of the housing market [14]. Prefabricated construction will likely become the focus of future development of the construction industry.

Prefabricated construction has been widely applied in many countries [15]. China has also actively developed prefabricated construction, but construction ratio and scale are still low due to their high cost [16]. Faced with the contradiction between the cost of prefabricated construction and market needs, the Chinese government [17] and provinces [18–20] have launched prefabricated construction subsidy policies. Common subsidy policies include subsidizing the assembler, the manufacturer, or consumers who buy prefabricated construction [47, 48]. Although government subsidies are currently an important driving force for the development of prefabricated construction, it also affects the manufacturer’s wholesale price, the assembler’s retail price, and the assembly rate. Subsidy policies for different subsidy objects have different impacts on the decision-making of the assembler and the manufacturer. The government subsidizes the assembler, the manufacturer, or consumers, thereby hoping to increase the assembly rate and lower the price, to promote the development of prefabricated construction. Therefore, when the government subsidizes the assembler or the manufacturer, the two parties want to obtain more government subsidies, and they need to increase the assembly rate; however, at the same time, doing so will increase the cost to the assembler and the manufacturer. In this case, the assembler and the manufacturer need to consider how to determine the wholesale price, the retail price, and the assembly rate to maximize their profit. When the government subsidizes consumers, the assembler and the manufacturer want to attract more consumers and increase demand through government subsidies; therefore, both parties need to increase the assembly rate. However, at the same time, doing so will increase the cost of the assembler and the manufacturer. In this case, the assembler and the manufacturer need to consider how to determine the wholesale price, the retail price, and the assembly rate to maximize their profit. Therefore, we mainly discuss the following two questions: (i) What is the manufacturer’s optimal wholesale price, the assembler’s optimal retail price, and the optimal assembly rate under different subsidy objects? (ii) Which subsidy method is better for the government, the assembler, the manufacturer, or customers?

This study aims to address these issues. Compared with previous research, to make this study more in line with the actual situation of the prefabricated construction supply chain, we innovatively introduce the assembly rate decision into the prefabricated construction supply chain, and assume that the assembler needs to determine the retail price and the assembly rate. In addition, according to the government’s policy of subsidizing different objects, we consider the three situations when the government subsidizes the assembler, the manufacturer and consumers. The results can provide relevant decision suggestions for the manufacturer and the assembler of a prefabricated construction supply chain under different subsidy policies, and provide policy suggestions for the government to formulate a prefabricated construction subsidy policy.

The remainder of this paper is organized as follows. In Section 2, we presents a literature review on previous related research. In Section 3, we present model descriptions and assumptions. In Section 4, we study the base model. In Section 5, we study the manufacturer’s wholesale price and assembler’s retail price and assembly rate decision under three different subsidy objects. We provide comparisons of the results of the three models with each other. Numerical studies are used to analyze the influence of different parameters on the manufacturer’s wholesale price and assembler’s retail price and assembly rate decision in Section 6. Section 7 concludes our study, and future research directions are explored.

## Literature review

Supply chain management has emerged as a popular and useful concept in both the construction industry and the research community since the mid-1990s [21]. Many scholars have widely recognized the significance of applying supply chain management in the construction industry [22–24]. Aloini et al. [25] investigated the development of supply chain management in the construction industry, adopted a literature review approach, and studied the risk factors affecting the implementation of supply chain management. Butkovi et al. [26] summarized the results with regard to the construction project subjects covered by existing construction supply chain studies, and the studies showed that most papers have focused their research on contractors’ decisions and suppliers’ decisions. Xue et al. [27] used multilevel planning theory to establish a two-level planning model with the goal of profit maximization. This model could focus on the profit maximization or cost minimization of all partners at different decision-making levels. Reza et al. [22] evaluated the construction supply chain using a mathematical model of biobjective linear programming and discussed the synergy between supplier selection and project planning and scheduling in the proposed green supply chain. Liu and Tao [28] presented a multiobjective purchasing model, that aimed to reduce the purchasing and supply costs, as well as the lead time in a construction supply chain.

Although there have been many studies on the construction supply chains, research on prefabricated construction supply chain management is still relatively limited [29]. Wang et al. [30] established a computational model to estimate the total cost of a prefabricated construction supply chain based on the activity-based costing (ABC) method, and studied the problem of predicting a supply chain cost for prefabricated construction under an uncertain situation. Yang et al. [31] concluded the ordering strategy on construction material based on the traditional EOQ model. Han et al. [32] established a comprehensive game model under component self-manufacturing and outsourcing, and analyzed the profit levels of manufacturers and contractors in the supply chain. Wu et al. [33] developed a theoretical transaction cost framework of a prefabricated construction supply chain based on an extensive literature review. Second, an empirical study was conducted on two cases in Chongqing to validate the transaction costs framework. Wang et al. [30] established a computational model to estimate the total cost of a prefabricated construction supply chain based on the activity-based costing method. The model can assist in finding the critical areas for cost reduction of the whole supply chain. Du et al. [34] applied structural equation modeling, and explored the key factors affecting carbon emissions and the influencing relationships from the perspective of the supply chain. The results confirmed that technical factors have the strongest effects on reducing carbon emissions in prefabricated building supply chains (PBSCs) and that supply chain coordination factors have the weakest effects. The above literature mainly studied the cost, ordering, and management of the prefabricated construction supply chain; however, there are relatively few studies on pricing.

To promote the rapid development of the industry, the government has introduced subsidy policies in many industries. Research has shown that government subsidies can improve enterprises’ production efficiency [35], but they also affect the decisions of supply chain members [36]. Common subsidy policies include direct subsidizes for enterprises. Xue et al. [37] studied the influences of government subsidies on retail prices, market demand, and green supply chain profit. The results showed that government subsidies can significantly improve social welfare and promote the improvement of energy-saving products. Su et al. [38] examined how to decide the production and price of green supply chain members in the case of consumers’ green preferences with different government subsidies. Based on the government subsidy function and Stackelberg model, Zhou and Wei [39] constructed a three-level green supply chain system that includes manufacturers, suppliers, and customers, and analyzed the incentive mechanism on the decision and profit of supply chain members. Other subsidy policies include subsidizes for consumers. Mohamed et al. [40] showed government economic compensation to be an important factor to encourage farmers to supply straw for biomass to energy conversion. Policy incentives can effectively improve the development of straw supply chain systems.

The Chinese government and provinces have introduced subsidy policies to promote the development of prefabricated construction. Research has shown that the government plays a dominant role in the promotion of prefabricated construction in China [41]. More incentive policies are required to be provided by the Chinese government [42, 43], such as financial support and tax incentives. Other researchers have also studied the impact of subsidies on the development of prefabricated construction [44, 45]. The above literature has mainly studied the impact of government subsidies on the development of prefabricated construction; thus, there is a lack of research on the impact of subsidies on the supply chain decisions of prefabricated construction.

The above literature on the prefabricated construction supply chain and enterprises’ pricing provided the basis for our study, which led us to decide to use the manufacturer’s pricing and the assembler’s pricing as variables to study the prefabricated construction supply chain. In addition, the above literature on the impact of subsidies on supply chain decisions notes out that different subsidy objects have an important impact on supply chain decisions and management. Therefore, we innovatively introduce the subsidy policy into the prefabricated construction supply chain, and study the decision of the prefabricated construction supply chain when the subsidy objects are different, which can better reflect the current situation of prefabricated construction.

## Model descriptions and assumptions

In this paper, we study the wholesale price, retail price, assembly rate, manufacturer profit, and the assembler profit under government subsidies. We describe a two-echelon prefabricated construction supply chain model that consists of a single manufacturer and a single assembler. The manufacturer produces prefabricated components, and takes the components required for a house as a unit. The assembler is responsible for assembling components and selling them to consumers. In the supply chain, we assume that the manufacturer is dominant and determine the wholesale price. The assembler is the follower and determines the retail price and the assembly rate. Different subsidy objects can be divided into three categories: subsidized the assembler, subsidized the manufacturer, and subsidized consumers. Therefore, we compared the optimal decision-making under three different subsidy objects to find the most suitable subsidy method for the government, the assembler, the manufacturer, or customers. The notations of the parameters and variables in this paper are presented in Table 1.

**Table 1:** Notations of parameters and variables.

Decision variables	Descriptions
***ω***	Manufacturer’s unit wholesale price.
***p***	Assembler’s unit retail price.
***δ***	Construction assembly rate.
**Parameters**	
***c***	Manufacturer’s unit cost.
***δ***	Lower limit of subsidy for assembly rate stipulated by the government.
***θ***	Unit product subsidy coefficient stipulated by the government.
***g***_**0**_	initial housing loans coefficient stipulated by the government.
***g***	Increasing housing loans coefficient stipulated by the government.
***ε***	Cost factor for increasing construction assembly rate.
***p***_***i***_ **(*ω*)**	Retail price when ***ω*** is not calculated, ***i*** = 1, 2, 3, 4. The values 1, 2, 3, and 4 represent the retail price when there is no government subsidy and the government subsidizes the assembler, the manufacturer and consumers, respectively.
***δ***_***i***_ **(*ω*)**	Construction assembly rate when ***ω*** is not calculated, ***i*** = 1, 2, 3, 4. The values 1, 2, 3, and 4 represent the assembly rate when there is no government subsidy and the government subsidizes the assembler, the manufacturer and consumers, respectively.
***D***_***i***_ **(*p*, *δ*)**	Demand when the government subsidizes the assembler, the manufacture and consumers, respectively, *i* = 1, 2.
***I*(*T***_***i***_ **)**	Subsidy function when the government subsidizes the manufacture or the assembler, respectively, *i* = 1, 2.

In addition, the assumptions in this study are as follows.

1. We assume that the demand function depends on the retail price (*p*), the assembly rate (*δ*), and the housing loans coefficient (*g*_0_). That is, *D*_1_(*p*, *δ*) = *α* − *βp* + *γδ* + *φg*_0_, where *α* represents the total market demand for prefabricated construction, and *β* represents the sensitivity of consumers to the retail price *p*. To describe the characteristics of demand, we introduce *γ* to express the impact of environmentally conscious consumers on demand [46]. *φ* represents the sensitivity of consumers to the housing loans coefficient. *α*, *β*, *γ*, *φ* are all greater than 0.
2. According to subsidy policies, the government subsidizes consumers by increasing the housing loan coefficient [47]. Doing so may attract more consumers and increase prefabricated construction demand. When the construction assembly rate is higher than the assembly rate subsidy floor (*δ* ≥ *δ*), the government increases the housing loan coefficient. That is *D*_2_(*p*, *δ*) = *α* − *βp* + *γδ* + *φg*.
3. According to subsidy policies, when enterprises adopt prefabricated construction, the government will give a certain percentage of financial rewards based on the incremental costs of enterprises [48]. When the construction assembly rate is higher than the lower limit of subsidy (*δ* ≥ *δ*), enterprises receive government subsidies. When the government subsidizes the assembler, it is shown as: $I\left(T_{1}\right)=\{\begin{array}{cc} \theta \omega \left(\alpha -\beta p+\gamma \delta +\varphi g_{0}\right) & \delta \geq \underline{\delta }\\ 0 & \delta <\underline{\delta } \end{array}$. When the government subsidizes the manufacturer, it is shown as: $I\left(T_{2}\right)=\{\begin{array}{cc} \theta c\left(\alpha -\beta p+\gamma \delta +\varphi g_{0}\right)\; & \delta \geq \underline{\delta }\\ 0 & \delta <\underline{\delta } \end{array}$.
4. The relationship between the assembler’s cost and the assembly rate is *μ*(*δ*) = *εδ*^2^. This assumption means that the assembler’s cost is a quadratic function of *δ*. This setting is popular in the literature [49].
5. To make the Hessian matrix in the basic model negatively definite, we assume that *γ*^2^ < 4*εβ*.

## Base model

In this section, we propose a base model without government subsidies. The manufacturer determines the wholesale price to maximize its profit, and the assembler determines the retail price and the assembly rate to maximize its profit.

According to the assumptions mentioned above, the manufacturer’s profit $\pi_{1}^{M}\left(\omega \right)$, is:

$$\pi_{1}^{M}\left(\omega \right)=\left(\omega -c\right)D_{1}\left(p,\delta \right)$$ (1)

The assembler’s profit $\pi_{1}^{A}\left(p,\delta \right)$, is:

$$\pi_{1}^{A}\left(p,\delta \right)=\left(p-\omega \right)D_{1}\left(p,\delta \right)-\mu \left(\delta \right)$$ (2)

The first term is the profit from selling prefabricated construction. The second term indicates the cost of increasing the assembly rate.

**Proposition 1**. In this section, the manufacturer’s optimal wholesale price (*ω*_1_) is:

$$\omega_{1}=\frac{\alpha +c\beta +\varphi g_{0}}{2\beta }$$ (3)

The assembler’s optimal retail price (*p*_1_) is:

$$p_{1}=\frac{6\varepsilon \beta \alpha +6\varepsilon \beta \varphi g_{0}+c\beta \left(2\varepsilon \beta -\gamma^{2}\right)-\gamma^{2}\left(\alpha +\varphi g_{0}\right)}{8\varepsilon \beta^{2}-2\beta \gamma^{2}}$$ (4)

The construction assembly rate (*δ*_1_) is:

$$\delta_{1}=\frac{\gamma \left(\alpha +\varphi g_{0}-\beta c\right)}{8\varepsilon \beta -2\gamma^{2}}$$ (5)

**Proof**. In the base decision model, from (2), $\frac{\partial \pi_{1}^{A}\left(p,\delta \right)}{\partial p}=\alpha -2\beta p+\gamma \delta +\beta \omega +\varphi g_{0},\;\frac{\partial \pi_{1}^{A}\left(p,\delta \right)}{\partial \delta }=\gamma \left(p-\omega \right)-2\varepsilon \delta$ can be got and $\frac{\partial^{2}\pi_{1}^{A}\left(p,\delta \right)}{\partial p^{2}}=-2\beta <0,\;\frac{\partial^{2}\pi_{1}^{A}\left(p,\delta \right)}{\partial \delta^{2}}=-2\varepsilon <0,\;\frac{\partial^{2}\pi_{1}^{A}\left(p,\delta \right)}{\partial p\partial \delta }=\frac{\partial^{2}\pi_{1}^{A}\left(p,\delta \right)}{\partial \delta \partial p}=\gamma$. when *γ*^2^ < 4*εβ*, $H\left(p,\delta \right)=\left|\begin{array}{cc} \frac{\partial^{2}\pi_{1}^{A}\left(p,\delta \right)}{\partial p^{2}} & \frac{\partial^{2}\pi_{1}^{A}\left(p,\delta \right)}{\partial p\partial \delta }\\ \frac{\partial^{2}\pi_{1}^{A}\left(p,\delta \right)}{\partial \delta \partial p} & \frac{\partial^{2}\pi_{1}^{A}\left(p,\delta \right)}{\partial \delta^{2}} \end{array}\right|=4\beta \varepsilon -\gamma^{2}>0$, we can see that $\pi_{1}^{A}\left(p,\delta \right)$ is a concave function, *p*_1_ and *δ*_1_ are always nonnegative, the maximal $\pi_{1}^{A}\left(p,\delta \right)$ exist and can be obtained from setting its first derivative equals 0. Hence, we have: $p_{1}\left(\omega \right)=\;\frac{2\varepsilon \left(\alpha +\varphi g_{0}\right)+\omega \left(2\varepsilon \beta -\gamma^{2}\right)}{4\varepsilon \beta -\gamma^{2}}$ and $\delta_{1}\left(\omega \right)=\frac{\gamma \left(\alpha -\beta \omega +\varphi g_{0}\right)}{4\varepsilon \beta -\gamma^{2}}$. Substitute *p*_1_(*ω*) and *δ*_1_(*ω*) into (1), we can get $\frac{\partial \pi_{1}^{M}\left(\omega \right)}{\partial \omega }=\frac{2\varepsilon \beta \left(\alpha -2\beta \omega +\beta c+\varphi g_{0}\right)}{\left(4\varepsilon \beta -\gamma^{2}\right)},\;\frac{\partial^{2}\pi_{1}^{M}\left(\omega \right)}{\partial \omega^{2}}=\frac{-4\varepsilon \beta^{2}}{\left(4\varepsilon \beta -\gamma^{2}\right)}<0$. $\pi_{1}^{M}\left(\omega \right)$ is a concave function, *ω*_1_ is always nonnegative, and the maximal $\pi_{1}^{M}\left(\omega \right)$ exists and can be obtained by setting its first derivative equal to 0. Hence, we can get (3). Substituting (3) into *p*_1_(*ω*) and *δ*_1_(*ω*), we can obtain (4) and (5). This completes the proof.

According to proposition 1, we can obtain the optimal wholesale price, the optimal retail price, and the optimal assembly rate in this situation. With the increase in customer preference, the assembly rate and the retail price increase. It can be explained that when *γ* is higher, the assembler will attract more customers by increasing the assembly rate, and the demand for prefabricated construction will also increase. However, while increasing the assembly rate, the cost of the assembler also increases; thus, the retail price will increase.

## Subsidy models

In this section, we discuss the models under three different subsidy objects: the government subsidizes the assembler, the manufacturer, or consumers. Then, we discuss the influence of different subsidy objects on manufacturer pricing, assembler pricing, assembly rate decision, manufacturer profit, and assembler profit.

### The model when the government subsidizes the assembler

In this section, the government subsidizes the assembler. First, the manufacturer determines the wholesale price based on costs and orders. Second, the assembler determines the retail price and the assembly rate based on the given wholesale price and government subsidy.

According to the assumptions mentioned above, the manufacturer’s profit $\pi_{2}^{M}\left(\omega \right)$, is:

$$\pi_{2}^{M}\left(\omega \right)=\left(\omega -c\right)D_{1}\left(p,\delta \right)$$ (6)

According to the assumptions mentioned above, the assembler’s profit $\pi_{2}^{A}\left(p,\delta \right)$, is:

$$\pi_{2}^{A}\left(p,\delta \right)=\left(p-\omega \right)D_{1}\left(p,\delta \right)+I\left(T_{1}\right)-\mu \left(\delta \right)$$ (7)

The first term is the profit from selling prefabricated construction. The second term indicates the government subsidy. The third term is the cost of increasing the assembly rate.

**Proposition 2**. The manufacturer’s optimal wholesale price (*ω*_2_) is:

$$\omega_{2}=\frac{\alpha +c\beta \left(1-\theta \right)+\varphi g_{0}}{2\beta \left(1-\theta \right)}$$ (8)

The assembler’s optimal retail price (*p*_2_) is:

$$p_{2}=\frac{6\varepsilon \beta \alpha +6\varepsilon \beta \varphi g_{0}+c\beta \left(1-\theta \right)\left(2\varepsilon \beta -\gamma^{2}\right)-\gamma^{2}\left(\alpha +\varphi g_{0}\right)}{8\varepsilon \beta^{2}-2\beta \gamma^{2}}$$ (9)

The construction assembly rate (*δ*_2_) is:

$$\delta_{2}=\frac{\gamma \left[\alpha -c\beta \left(1-\theta \right)+\varphi g_{0}\right]}{8\varepsilon \beta -2\gamma^{2}}$$ (10)

**Proof**. From (7), $\frac{\partial \pi_{2}^{A}\left(p,\delta \right)}{\partial p}=\alpha -2\beta p+\gamma \delta +\varphi g_{0}+\beta \omega \left(1-\theta \right),\;\frac{\partial \pi_{2}^{A}\left(p,\delta \right)}{\partial \delta }=\gamma \left[p-\omega \left(1-\theta \right)\right]-2\varepsilon \delta$ can be got and $\frac{\partial^{2}\pi_{2}^{A}\left(p,\delta \right)}{\partial p^{2}}=-2\beta <0,\;\frac{\partial^{2}\pi_{2}^{A}\left(p,\delta \right)}{\partial \delta^{2}}=-2\varepsilon <0,\;\frac{\partial^{2}\pi_{2}^{A}\left(p,\delta \right)}{\partial p\partial \delta }=\frac{\partial^{2}\pi_{2}^{A}\left(p,\delta \right)}{\partial \delta \partial p}=\gamma$. when *γ*^2^ < 4*εβ*, $H\left(p,\delta \right)=\left|\begin{array}{cc} \frac{\partial^{2}\pi_{2}^{A}\left(p,\delta \right)}{\partial p^{2}} & \frac{\partial^{2}\pi_{2}^{A}\left(p,\delta \right)}{\partial p\partial \delta }\\ \frac{\partial^{2}\pi_{2}^{A}\left(p,\delta \right)}{\partial \delta \partial p} & \frac{\partial^{2}\pi_{2}^{A}\left(p,\delta \right)}{\partial \delta^{2}} \end{array}\right|=4\beta \varepsilon -\gamma^{2}>0$. we can see that $\pi_{2}^{A}\left(p,\delta \right)$ is a concave function, *p*_2_ and *δ*_2_ are always nonnegative, the maximal $\pi_{2}^{A}\left(p,\delta \right)$ exist and can be obtained from setting its first derivative equals 0. Hence, we have: $p_{2}\left(\omega \right)=\frac{2\varepsilon \left(\alpha +\varphi g_{0}\right)+\omega \left(1-\theta \right)\left(2\varepsilon \beta -\gamma^{2}\right)}{4\varepsilon \beta -\gamma^{2}}$ and $\delta_{2}\left(\omega \right)=\frac{\gamma \left[\alpha -\omega \beta \left(1-\theta \right)+\varphi g_{0}\right]}{4\varepsilon \beta -\gamma^{2}}$. Substitute *p*_2_(*ω*) and *δ*_2_(*ω*) into (6), we can get $\frac{\partial \pi_{2}^{M}\left(\omega \right)}{\partial \omega }=\frac{2\varepsilon \beta \left(\alpha -2\omega \beta +2\theta \omega \beta -\theta c\beta +c\beta \right)}{\left(4\varepsilon \beta -\gamma^{2}\right)},\;\frac{\partial^{2}\pi_{2}^{M}\left(\omega \right)}{\partial \omega^{2}}=\frac{-4\varepsilon \beta^{2}\left(1-\theta \right)}{\left(4\varepsilon \beta -\gamma^{2}\right)}<0$. $\pi_{2}^{M}\left(\omega \right)$ is a concave function, *ω*_2_ is always nonnegative, and the maximal $\pi_{2}^{M}\left(\omega \right)$ exists and can be obtained by setting its first derivative equal to 0. Hence, we can get (8), Substituting (8) into *p*_2_(*ω*) and *δ*_2_(*ω*), we can obtain (9) and (10). This completes the proof.

From Proposition 2, we can obtain the optimal wholesale price, the optimal retail price, and the optimal assembly rate in this situation. The wholesale price and the assembly rate increase with increasing subsidy coefficient, and the retail price decreases with increasing subsidy coefficient. It can be explained that government subsidies can bring additional profits to the assembler. When *θ* is higher, the assembler will receive more subsidies. Therefore, the assembler is willing to lower the retail price and increase the assembly rate. To obtain more profits, the manufacturer will increase the wholesale price.

**Proposition 3**. To compare with the base model, we have the following results: (1) *ω*_2_ > *ω*_1_; (2) when $\varepsilon >\frac{\gamma^{2}}{2\beta }$, *p*_2_ > *p*_1_, when $\varepsilon <\frac{\gamma^{2}}{2\beta }$, *p*_2_ > *p*_1_; and (3) *δ*_2_ < *δ*_1_; (4) $\pi_{2}^{M}\left(\omega \right)>\pi_{1}^{M}\left(\omega \right)$; (5) $\pi_{2}^{A}\left(p,\;\delta \right)>\pi_{1}^{A}\left(p,\;\delta \right)$.

**Proof**. We have $\omega_{2}-\omega_{1}=\frac{\theta \left(\alpha +\varphi g_{0}\right)}{2\beta \left(1-\theta \right)}>0$. $p_{2}-p_{1}=-\frac{\theta c\beta \left(2\varepsilon \beta -\gamma^{2}\right)}{8\varepsilon \beta^{2}-2\beta \gamma^{2}}$, when $\varepsilon >\frac{\gamma^{2}}{2\beta }$, *p*_2_ < *p*_1_; when $\varepsilon <\frac{\gamma^{2}}{2\beta }$, *p*_2_ > *p*_1_. We have $\delta_{2}-\delta_{1}=\frac{\theta \gamma \beta c}{8\varepsilon \beta -2\gamma^{2}}>0$, so we can get *δ*_2_ > *δ*_1_. Substitute (3), (4) and (5) into (1) and (2), we can get $\pi_{1}^{M}\left(\omega \right)=\frac{\varepsilon {\left(\alpha +\varphi g_{0}-\beta c\right)}^{2}}{8\varepsilon \beta -2\gamma^{2}}$ and $\pi_{1}^{A}\left(p,\;\delta \right)=\frac{\varepsilon {\left(\alpha +\varphi g_{0}-\beta c\right)}^{2}}{4\left(4\varepsilon \beta -\gamma^{2}\right)}$. Substitute (8), (9) and (10) into (6) and (7), we can get $\pi_{2}^{M}\left(\omega \right)=\frac{\varepsilon {\left[\alpha -\beta c\left(1-\theta \right)+\varphi g_{0}\right]}^{2}}{\left(1-\theta \right)\left(8\varepsilon \beta -2\gamma^{2}\right)}$ and $\pi_{2}^{A}\left(p,\;\delta \right)=\frac{\varepsilon {\left[\alpha -\beta c\left(1-\theta \right)+\varphi g_{0}\right]}^{2}}{4\left(4\varepsilon \beta -\gamma^{2}\right)}$. We have [*α* − *βc*(1 − *θ*) + *φg*_0_] − (*α* − *βc* + *φg*_0_) = *θβc* > 0, we can get $\pi_{2}^{M}\left(\omega \right)>\pi_{1}^{M}\left(\omega \right)$ and $\pi_{2}^{A}\left(p,\delta \right)>\pi_{1}^{A}\left(p,\delta \right)$. This completes the proof.

This proposition indicates that, with subsidizing the assembler situation, the assembler will reduce the retail price and increase the assembly rate. To obtain more profits, the manufacturer will increase the wholesale price. Although the assembler’s cost has increased, both parties can obtain more profits. This is beneficial to both parties and the development of prefabricated construction.

### The model when the government subsidizes the manufacturer

In this section, the government subsidizes the manufacturer. First, the manufacturer determines the wholesale price based on costs, orders and government subsidies. Second, the assembler determines the retail price and the assembly rate based on the given wholesale price.

According to the assumptions mentioned above, the manufacturer’s profit $\pi_{3}^{M}\left(\omega \right)$, is:

$$\pi_{3}^{M}\left(\omega \right)=\left(\omega -\text{c}\right)D_{1}\left(p,\delta \right)+I\left(T_{2}\right)$$ (11)

The first term is the profit from selling prefabricated construction. The second term indicates the government subsidy.

The assembler’s profit $\pi_{3}^{A}\left(p,\delta \right)$, is:

$$\pi_{3}^{A}\left(p,\delta \right)=\left(p-\omega \right)D_{1}\left(p,\delta \right)-\mu \left(\delta \right)$$ (12)

The first term is the profit from selling prefabricated construction. The second term indicates the cost of increasing the construction assembly rate.

**Proposition 4**. The manufacturer’s optimal wholesale price (*ω*_3_) is:

$$\omega_{3}=\frac{\alpha +c\beta \left(1-\theta \right)+\varphi g_{0}}{2\beta }$$ (13)

The assembler’s optimal retail price (*p*_3_) is:

$$p_{3}=\frac{6\varepsilon \beta \alpha +6\varepsilon \beta \varphi g_{0}+c\beta \left(1-\theta \right)\left(2\varepsilon \beta -\gamma^{2}\right)-\gamma^{2}\left(\alpha +\varphi g_{0}\right)}{8\varepsilon \beta^{2}-2\beta \gamma^{2}}$$ (14)

The construction assembly rate (*δ*_3_) is:

$$\delta_{3}=\frac{\gamma \left[\alpha -c\beta \left(1-\theta \right)+\varphi g_{0}\right]}{8\varepsilon \beta -2\gamma^{2}}$$ (15)

**Proof**. From (12), $\frac{\partial \pi_{3}^{A}\left(p,\delta \right)}{\partial p}=\alpha -2\beta p+\gamma \delta +\beta \omega +\varphi g_{0},\;\frac{\partial \pi_{3}^{A}\left(p,\delta \right)}{\partial \delta }=\gamma \left(p-\omega \right)-2\varepsilon \delta$ can be got and $\frac{\partial^{2}\pi_{3}^{A}\left(p,\delta \right)}{\partial p^{2}}=-2\beta <0,\;\frac{\partial^{2}\pi_{3}^{A}\left(p,\delta \right)}{\partial \delta^{2}}=-2\varepsilon <0,\;\frac{\partial^{2}\pi_{3}^{A}\left(p,\delta \right)}{\partial p\partial \delta }=\frac{\partial^{2}\pi_{3}^{A}\left(p,\delta \right)}{\partial \delta \partial p}=\gamma$. when *γ*^2^ < 4*εβ*, $H\left(p,\delta \right)=\left|\begin{array}{cc} \frac{\partial^{2}\pi_{3}^{A}\left(p,\delta \right)}{\partial p^{2}} & \frac{\partial^{2}\pi_{3}^{A}\left(p,\delta \right)}{\partial p\partial \delta }\\ \frac{\partial^{2}\pi_{3}^{A}\left(p,\delta \right)}{\partial \delta \partial p} & \frac{\partial^{2}\pi_{3}^{A}\left(p,\delta \right)}{\partial \delta^{2}} \end{array}\right|=4\beta \varepsilon -\gamma^{2}>0$. we can see that $\pi_{3}^{A}\left(p,\delta \right)$ is a concave function, *p*_3_ and *δ*_3_ are always nonnegative, the maximal $\pi_{3}^{A}\left(p,\delta \right)$ exist and can be obtained from setting its first derivative equals 0. Hence, we have: $p_{3}\left(\omega \right)=\frac{2\varepsilon \left(\alpha +\varphi g_{0}\right)+\omega \left(2\varepsilon \beta -\gamma^{2}\right)}{4\varepsilon \beta -\gamma^{2}}$ and $\delta_{3}\left(\omega \right)=\frac{\gamma \left(\alpha -\beta \omega +\varphi g_{0}\right)}{4\varepsilon \beta -\gamma^{2}}$. Substitute *p*_3_(*ω*) and *δ*_3_(*ω*) into (11), we can get $\frac{\partial \pi_{3}^{M}\left(\omega \right)}{\partial \omega }=\frac{2\varepsilon \beta \left(\alpha -2\beta \omega +\beta c-\theta \beta \omega +\varphi g_{0}\right)}{\left(4\varepsilon \beta -\gamma^{2}\right)},\;\frac{\partial^{2}\pi_{3}^{M}\left(\omega \right)}{\partial \omega^{2}}=\frac{-4\varepsilon \beta^{2}}{\left(4\varepsilon \beta -\gamma^{2}\right)}<0$. $\pi_{3}^{M}\left(\omega \right)$ is a concave function, *ω*_3_ is always nonnegative, and the maximal $\pi_{3}^{M}\left(\omega \right)$ exists and can be obtained by setting its first derivative equal to 0. Hence, we can get (13), Substituting (13) into *p*_3_(*ω*) and *δ*_3_(*ω*), we can obtain (14) and (15). This completes the proof.

From Proposition 4, we can obtain the optimal wholesale price, the optimal retail price, and the optimal assembly rate in this situation. The wholesale price and the retail price decrease with increasing the subsidy coefficient, and the assembly rate increases with increasing the subsidy coefficient. It can be explained that government subsidies can bring additional profits to the manufacturer. When the subsidy coefficient is higher, the manufacturer will receive more subsidies. The manufacturer lowers the wholesale price, and the cost to the assembler would also be reduced. Therefore, the assembler will lower the retail price and increase the assembly rate.

**Proposition 5**. To compare with the base mode, we have the following results: (1) *ω*_3_ < *ω*_1_; (2) when $\varepsilon >\frac{\gamma^{2}}{2\beta }$, *p*_3_ < *p*_1_, when $\varepsilon <\frac{\gamma^{2}}{2\beta }$, *p*_3_ > *p*_1_; (3) *δ*_3_ > *δ*_1_; (4) $\pi_{3}^{M}\left(\omega \right)>\pi_{1}^{M}\left(\omega \right)$; (5) $\pi_{3}^{A}\left(p,\delta \right)>\pi_{1}^{A}\left(p,\delta \right)$.

**Proof**. $\omega_{3}-\omega_{1}=-\frac{\theta c\beta }{2\beta }<0$, we can get *ω*_3_ < *ω*_1_. $p_{3}-p_{1}=-\frac{\theta c\beta \left(2\varepsilon \beta -\gamma^{2}\right)}{8\varepsilon \beta^{2}-2\beta \gamma^{2}}$, when $\varepsilon >\frac{\gamma^{2}}{2\beta }$, *p*_3_ < *p*_1_; when $\varepsilon <\frac{\gamma^{2}}{2\beta }$, *p*_3_ > *p*_1_. We have $\delta_{3}-\delta_{1}=\frac{\theta \gamma \beta c}{8\varepsilon \beta -2\gamma^{2}}>0$, so we can get *δ*_3_ > *δ*_1_. Substitute (13), (14) and (15) into (11) and (12), we can get $\pi_{3}^{M}\left(\omega \right)=\frac{\varepsilon {\left[\alpha -\beta c\left(1-\theta \right)+\varphi g_{0}\right]}^{2}}{8\varepsilon \beta -2\gamma^{2}}$ and $\pi_{3}^{A}\left(p,\delta \right)=\frac{\varepsilon {\left[\alpha -\beta c\left(1-\theta \right)+\varphi g_{0}\right]}^{2}}{4\left(4\varepsilon \beta -\gamma^{2}\right)}$. We have [*α* − *βc*(1 − *θ*) + *φg*_0_] − (*α* − *βc* + *φg*_0_) = *θβc* > 0, we can get $\pi_{3}^{M}\left(\omega \right)>\pi_{1}^{M}\left(\omega \right)$ and $\pi_{3}^{A}\left(p,\delta \right)>\pi_{1}^{A}\left(p,\delta \right)$. This completes the proof.

This proposition indicates that by subsidizing the manufacturer situation, the manufacturer will lower the wholesale price, which reduces the cost of the assembler. Therefore, the assembler will lower the retail price and increase the assembly rate. Both the assembler and the manufacturer can obtain more profits. This is beneficial to both parties and the development of prefabricated construction.

### The model when the government subsidizes customers

In this section, the government subsidizes customers, which can attract more consumers and lead to change in demand. First, the manufacturer determines the wholesale price based on costs and orders. Second, the assembler determines the retail price and the assembly rate based on the given wholesale price.

According to the assumptions mentioned above, the manufacturer’s profit $\pi_{4}^{M}\left(\omega \right)$, is:

$$\pi_{4}^{M}\left(\omega \right)=\left(\omega -c\right)D_{2}\left(p,\delta \right)$$ (16)

The assembler’s profit $\pi_{4}^{A}\left(p,\delta \right)$, is:

$$\pi_{4}^{A}\left(p,\delta \right)=\left(p-\omega \right)D_{2}\left(p,\delta \right)-\mu \left(\delta \right)$$ (17)

The first term is the profit from selling prefabricated construction. The second term indicates the cost of increasing the construction assembly rate.

**Proposition 6**. The manufacturer’s optimal wholesale price (*ω*_4_) is:

$$\omega_{4}=\frac{\alpha +c\beta +\varphi g}{2\beta }$$ (18)

The assembler’s optimal retail price (*p*_4_) is:

$$p_{4}=\frac{6\varepsilon \beta \alpha +6\varepsilon \beta \varphi g+c\beta \left(2\varepsilon \beta -\gamma^{2}\right)-\gamma^{2}\left(\alpha +\varphi g\right)}{8\varepsilon \beta^{2}-2\beta \gamma^{2}}$$ (19)

The construction assembly rate (*δ*_4_) is:

$$\delta_{4}=\frac{\gamma \left(\alpha +\varphi g-\beta c\right)}{8\varepsilon \beta -2\gamma^{2}}$$ (20)

**Proof**. From (17), $\frac{\partial \pi_{4}^{A}\left(p,\delta \right)}{\partial p}=\alpha -2\beta p+\gamma \delta +\varphi g+\beta \omega ,\;\frac{\partial \pi_{4}^{A}\left(p,\delta \right)}{\partial \delta }=\gamma \left(p-\omega \right)-2\varepsilon \delta$ can be got and $\frac{\partial^{2}\pi_{4}^{A}\left(p,\delta \right)}{\partial p^{2}}=-2\beta <0,\;\frac{\partial^{2}\pi_{4}^{A}\left(p,\delta \right)}{\partial \delta^{2}}=-2\varepsilon <0,\;\frac{\partial^{2}\pi_{4}^{A}\left(p,\delta \right)}{\partial p\partial \delta }=\frac{\partial^{2}\pi_{4}^{A}\left(p,\delta \right)}{\partial \delta \partial p}=\gamma$. when *γ*^2^ < 4*εβ*, $H\left(p,\delta \right)=\left|\begin{array}{cc} \frac{\partial^{2}\pi_{4}^{A}\left(p,\delta \right)}{\partial p^{2}} & \frac{\partial^{2}\pi_{4}^{A}\left(p,\delta \right)}{\partial p\partial \delta }\\ \frac{\partial^{2}\pi_{4}^{A}\left(p,\delta \right)}{\partial \delta \partial p} & \frac{\partial^{2}\pi_{4}^{A}\left(p,\delta \right)}{\partial \delta^{2}} \end{array}\right|=4\beta \varepsilon -\gamma^{2}$. we can see that $\pi_{4}^{A}\left(p,\delta \right)$ is a concave function, *p*_4_ and *δ*_4_ are always nonnegative, the maximal $\pi_{4}^{A}\left(p,\delta \right)$ exist and can be obtained from setting its first derivative equals 0. Hence, we have: $p_{4}\left(\omega \right)=\frac{2\varepsilon \alpha +2\varepsilon \varphi g+\omega \left(2\varepsilon \beta -\gamma^{2}\right)}{4\varepsilon \beta -\gamma^{2}}$ and $\delta_{4}\left(\omega \right)=\frac{\gamma \left(\alpha +\varphi g-\beta \omega \right)}{4\varepsilon \beta -\gamma^{2}}$. Substitute *p*_4_(*ω*) and *δ*_4_(*ω*) into (16), we can get $\frac{\partial \pi_{4}^{M}\left(\omega \right)}{\partial \omega }=\frac{2\varepsilon \beta \left(\alpha +\varphi g+c\beta -2\omega \beta \right)}{\left(4\varepsilon \beta -\gamma^{2}\right)},\;\frac{\partial^{2}\pi_{4}^{M}\left(\omega \right)}{\partial \omega^{2}}=\frac{-4\varepsilon \beta^{2}}{\left(4\varepsilon \beta -\gamma^{2}\right)}<0$. $\pi_{4}^{M}\left(\omega \right)$ is a concave function, *ω*_4_ is always nonnegative, and the maximal $\pi_{4}^{M}\left(\omega \right)$ exists and can be obtained by setting its first derivative equal to 0. Hence, we can get (18), Substituting (18) into *p*_4_(*ω*) and *δ*_4_(*ω*), we can obtain (19) and (20). This completes the proof.

From Proposition 6, we can obtain the supply chain’s optimal decision in this situation. The wholesale price, retail price, and assembly rate increase with increasing the housing loan coefficient. It can be explained that when the housing loan coefficient is higher, more consumers are attracted, and the demand will increase accordingly. To meet the needs of consumers, the assembler will increase the assembly rate, but the assembler’s cost will also increase. To obtain more profits, the manufacturer will increase the wholesale price. However these will increase the assembler’s cost. so the assembler will increase the retail price considering its own profit.

**Proposition 7**. To compare with the base mode, we have the following results: (1) *ω*_4_ > *ω*_1_; (2) *p*_4_ > *p*_1_; (3) *δ*_4_ > *δ*_1_; (4) $\pi_{4}^{M}\left(\omega \right)>\pi_{1}^{M}\left(\omega \right)$; and (5) $\pi_{4}^{A}\left(p,\delta \right)>\pi_{1}^{A}\left(p,\delta \right)$.

**Proof**. $\omega_{4}-\omega_{1}=\frac{\varphi \left(g-g_{0}\right)}{2\beta }>0$, we can get *ω*_4_ > *ω*_1_. $p_{4}-p_{1}=\frac{\varphi \left(g-g_{0}\right)\left(6\varepsilon \beta -\gamma^{2}\right)}{8\varepsilon \beta^{2}-2\beta \gamma^{2}}>0$, we can get *p*_4_ > *p*_1_. $\delta_{4}-\delta_{1}=\frac{\gamma \varphi \left(g-g_{0}\right)}{8\varepsilon \beta -2\gamma^{2}}>0$, we can get *δ*_4_ > *δ*_1_. Substitute (18), (19) and (20) into (16) and (17), we can get $\pi_{4}^{M}\left(\omega \right)=\frac{\varepsilon {\left(\alpha +\varphi g-\beta c\right)}^{2}}{8\varepsilon \beta -2\gamma^{2}}$ and $\pi_{4}^{A}\left(p,\;\delta \right)=\frac{\varepsilon {\left(\alpha +\varphi g-\beta c\right)}^{2}}{4\left(4\varepsilon \beta -\gamma^{2}\right)}$. We have (*α* + *φg* − *βc*) − (*α* + *φg*_0_ − *βc*) = *φg* > 0, we can get $\pi_{4}^{M}\left(\omega \right)>\pi_{1}^{M}\left(\omega \right)$ and $\pi_{4}^{A}\left(p,\delta \right)>\pi_{1}^{A}\left(p,\delta \right)$. This completes the proof.

Proposition 7 shows that when the government subsidizes consumers, it can attract more consumers, and increase demand. When consumers receive government subsidies, the assembler needs to increase the assembly rate, but this will increase the assembler’s own cost. Therefore, the assembler will increase the retail price. To obtain more profits, the manufacturer will increase the wholesale price. Although the cost to the assembler has increased, both parties can obtain more profits. This is beneficial to both parties and the development of prefabricated construction.

**Proposition 8**. Comparing the wholesale price (*ω*), retail price (*p*), and assembly rate (*δ*), we obtain the following results:

(1) When $\theta >\frac{\varphi g-\varphi g_{0}}{\alpha +\varphi g}$, *ω*_2_ > *ω*_4_ > *ω*_3_; *p*_4_ > *p*_3_ = *p*_2_; *δ*_2_ = *δ*_3_ > *δ*_4_.
(2) When $\theta <\frac{\varphi g-\varphi g_{0}}{\alpha +\varphi g}$, *ω*_4_ > *ω*_2_ > *ω*_3_; *p*_4_ > *p*_3_ = *p*_2_; *δ*_4_ > *δ*_2_ = *δ*_3_.
(3) When $\theta =\frac{\varphi g-\varphi g_{0}}{\alpha +\varphi g}$, *ω*_2_ = *ω*_4_ > *ω*_3_; *p*_4_ > *p*_3_ = *p*_2_; *δ*_2_ = *δ*_3_ > *δ*_4_.

**Proof**. $\omega_{2}-\omega_{3}=\frac{\theta \left[\alpha +c\beta \left(1-\theta \right)+\varphi g_{0}\right]}{2\beta \left(1-\theta \right)}>0,\;\omega_{2}-\omega_{4}=\frac{\theta \alpha -\varphi g\left(1-\theta \right)+\varphi g_{0}}{2\beta \left(1-\theta \right)},\;\omega_{3}-\omega_{4}=\frac{\varphi g_{0}-\theta c\beta -\varphi g}{2\beta }<0$, when $\theta >\frac{\varphi g-\varphi g_{0}}{\alpha +\varphi g}$, *ω*_2_ > *ω*_4_ > *ω*_3_, when $\theta <\frac{\varphi g-\varphi g_{0}}{\alpha +\varphi g}$, *ω*_4_ > *ω*_2_ > *ω*_3_. *p*_2_ = *p*_3_, $p_{2}-p_{4}/p_{3}-p_{4}=-\frac{\theta c\beta \left(2\varepsilon \beta -\gamma^{2}\right)}{8\varepsilon \beta^{2}-2\beta \gamma^{2}}<0$, we can get *p*_4_ > *p*_3_ = *p*_2_. *δ*_2_ = *δ*_3_, $\delta_{2}-\delta_{4}/\delta_{3}-\delta_{4}=\gamma \frac{\theta \beta c+\varphi g_{0}-\varphi g}{8\varepsilon \beta -2\gamma^{2}}$, when $\theta >\frac{\varphi g-\varphi g_{0}}{\beta c}$, *δ*_2_ = *δ*_3_ > *δ*_4_, when $\theta <\frac{\varphi g-\varphi g_{0}}{\beta c}$, *δ*_4_ > *δ*_2_ = *δ*_3_. This completes the proof.

From propositions 8(1) and 8(3), it can be concluded that when $\theta >\frac{\varphi g-\varphi g_{0}}{\alpha +\varphi g}$ or $\theta =\frac{\varphi g-\varphi g_{0}}{\alpha +\varphi g}$, it is better for the government and customers to subsidize the manufacturer or the assembler. First, the government can lower the retail price and increase the assembly rate. Second, consumers are more concerned about the retail price. Lowering the retail price can attract more consumers and increase demand. This is more conducive to the promotion and development of prefabricated construction.

From proposition 8(2), it can be concluded that when $\theta <\frac{\varphi g-\varphi g_{0}}{\alpha +\varphi g}$, it is better for customers to subsidize the assembler or the manufacturer. Although in this case, the assembly rate is not the largest, it has also improved compared with the situation without government subsidies. Consumers can obtain a lower retail price, which can increase the demand for prefabricated construction. This is more conducive to the promotion and development of prefabricated construction.

**Proposition 9**. Comparing the manufacturer’s profit (*π*^*M*^(*ω*)) and the assembler’s profit (*π*^*A*^(*p*, *δ*)) when the government subsidizes different objects, we obtain the following results:

(1) when $\theta >\frac{\varphi g-\varphi g_{0}}{\beta c},\;\pi_{2}^{M}\left(\omega \right)>\pi_{3}^{M}\left(\omega \right)>\pi_{4}^{M}\left(\omega \right);\;\pi_{2}^{A}\left(p,\;\delta \right)=\pi_{3}^{A}\left(p,\;\delta \right)>\pi_{4}^{A}\left(p,\;\delta \right)$.
(2) when $\theta <\frac{\varphi g-\varphi g_{0}}{\beta c},\;\pi_{4}^{M}\left(\omega \right)>\pi_{2}^{M}\left(\omega \right)>\pi_{3}^{M}\left(\omega \right);\;\pi_{4}^{A}\left(p,\delta \right)>\pi_{2}^{A}\left(p,\delta \right)=\pi_{3}^{A}\left(p,\delta \right)$
(3) when $\theta =\frac{\varphi g-\varphi g_{0}}{\beta c},\;\pi_{2}^{M}\left(\omega \right)>\pi_{3}^{M}\left(\omega \right)=\pi_{4}^{M}\left(\omega \right),\;\pi_{2}^{A}\left(p,\delta \right)=\pi_{3}^{A}\left(p,\delta \right)=\pi_{4}^{A}\left(p,\delta \right)$

**Proof**. $\pi_{2}^{M}\left(\omega \right)-\pi_{3}^{M}\left(\omega \right)=\frac{\theta \varepsilon {\left[\alpha -\beta c\left(1-\theta \right)+\varphi g_{0}\right]}^{2}}{\left(1-\theta \right)\left(8\varepsilon \beta -2\gamma^{2}\right)}>0,\;\frac{\pi_{2}^{M}\left(\omega \right)}{\pi_{4}^{M}\left(\omega \right)}=\frac{\varepsilon {\left[\alpha -\beta c\left(1-\theta \right)+\varphi g_{0}\right]}^{2}}{\varepsilon {\left(\alpha +\varphi g-\beta c\right)}^{2}},\;\pi_{3}^{M}\left(\omega \right)-\pi_{4}^{M}\left(\omega \right)=\varepsilon \frac{{\left[\alpha +\varphi g_{0}-\beta c\left(1-\theta \right)\right]}^{2}-{\left(\alpha +\varphi g-\beta c\right)}^{2}}{8\varepsilon \beta -2\gamma^{2}}$, when $\theta >\frac{\varphi g-\varphi g_{0}}{\beta c},\;\pi_{2}^{M}\left(\omega \right)>\pi_{3}^{M}\left(\omega \right)>\pi_{4}^{M}\left(\omega \right)$, when $\theta <\frac{\varphi g-\varphi g_{0}}{\beta c},\;\pi_{4}^{M}\left(\omega \right)>\pi_{2}^{M}\left(\omega \right)>\pi_{3}^{M}\left(\omega \right)$. $\pi_{2}^{A}\left(p,\delta \right)=\pi_{3}^{A}\left(p,\delta \right)$, $\pi_{2}^{A}\left(p,\delta \right)-\pi_{4}^{A}\left(p,\delta \right)/\pi_{3}^{A}\left(p,\delta \right)-\pi_{4}^{A}\left(p,\delta \right)=\varepsilon \frac{{\left[\alpha -\beta c\left(1-\theta \right)+\varphi g_{0}\right]}^{2}-{\left(\alpha +\varphi g-\beta c\right)}^{2}}{4\left(4\varepsilon \beta -\gamma^{2}\right)}$, when $\theta >\frac{\varphi g-\varphi g_{0}}{\beta c},\;\pi_{2}^{A}\left(p,\delta \right)=\pi_{3}^{A}\left(p,\delta \right)>\pi_{4}^{A}\left(p,\delta \right)$, when $\theta <\frac{\varphi g-\varphi g_{0}}{\beta c},\;\pi_{4}^{A}\left(p,\delta \right)>\pi_{2}^{A}\left(p,\delta \right)=\pi_{3}^{A}\left(p,\delta \right)$. This completes the proof.

From propositions 9(1) and 9(3), it can be concluded that when $\theta >\frac{\varphi g-\varphi g_{0}}{\beta c}$, it is better for the assembler and the manufacturer to subsidize the assembler. Because the assembler and the manufacturer can obtain more profits, they will be willing to promote prefabricated construction. From proposition 9(2), it can be concluded that when $\theta <\frac{\varphi g-\varphi g_{0}}{\beta c}$, it is better for the assembler and the manufacturer to subsidize customers. Because they can obtain more profits, and they will be willing to promote prefabricated construction.

## Numerical analysis

This section compares the performances of different models: the base model, the model when the government subsidizes the assembler, the model when the government subsidizes the manufacturer, and the model when the government subsidizes customers. We discuss the impact of government subsidies on the manufacturer’s wholesale price, manufacturer’s profit, assembler’s retail price, assembly rate and assembler’s profit. We set *α* = 60, *β* = 1.35, *γ* = 0.64, *c* = 10, *ε* = 1.5 [50]. According to experience, *φ* = 1, *g*_0_ = 0.3, *g* = 0.5 [51]. To observe the change in decision variables, the assembler’s profit and the manufacturer’s profit, we set *θ* ∈ (0, 1]. The support data for this section is saved in the first set of data of S1 File. We also tested two sets of data within the range of values, and the conclusions are still applicable, but for the sake of brevity of the paper, we only put the first set of data in the paper, and the other two sets of data are also saved in S1 File.

### The influence of the subsidy coefficient on the wholesale price

**Fig 1:**
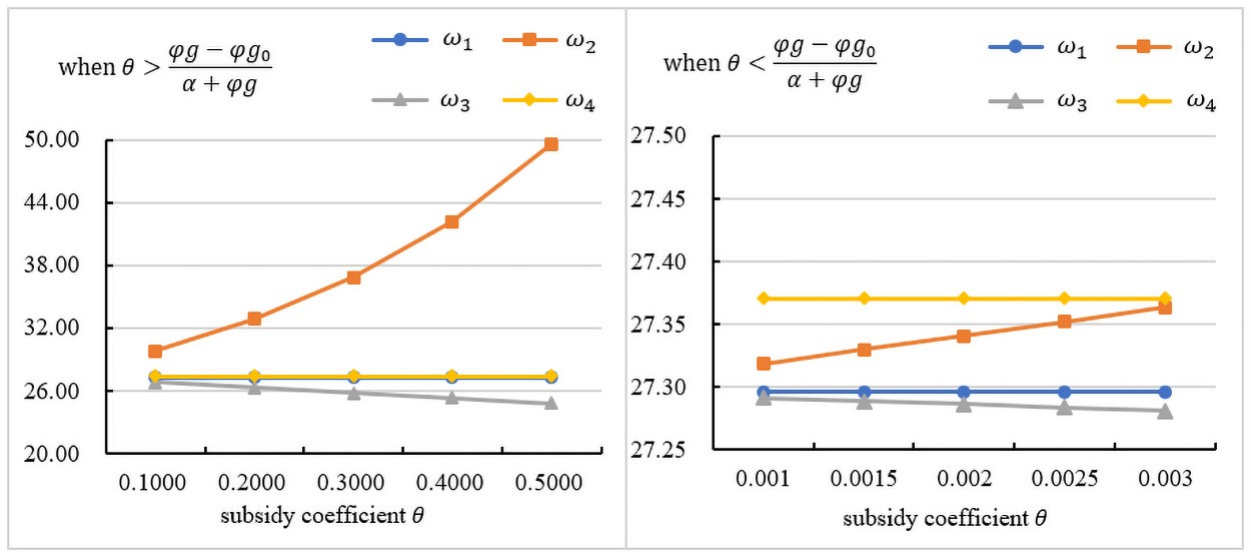
The influence of the subsidy coefficient on the wholesale price.

Fig 1 indicates the impact of *θ* on the wholesale price under different subsidy objects. As seen in the figures, when the government directly subsidizes the manufacturer, the wholesale price is the lowest, and it is inversely proportional to *θ*. Because the government subsidy can bring additional profits to the manufacturer, and under the restriction of the subsidy policy, the manufacturer is willing to lower the wholesale price. Lowering the wholesale price will reduce the cost of the assembler, thereby reducing the retail price. According to *D*_1_(*p*, *δ*), the demand will increase. According to *I*(*T*_2_), the manufacturer can obtain more subsidies. When *θ* is larger, the manufacturer can also obtain more subsidies. Therefore, when the government directly subsidizes the manufacturer, the wholesale price is the lowest. Fig 1(a) presents the trend in the wholesale price under $\theta >\frac{\varphi g-\varphi g_{0}}{\alpha +\varphi g}$. As seen in the figures, when the government subsidizes the assembler, the wholesale price is the highest, and it is proportional to *θ*. Fig 1(b) presents the trend in the wholesale price under $\theta <\frac{\varphi g-\varphi g_{0}}{\alpha +\varphi g}$. As seen in the figures, when the government subsidizes customers, the wholesale price is the highest. The manufacturer sets the wholesale price by maximizing its utility. Therefore, when the government subsidizes other objects, the manufacturer will increase the wholesale price to obtain more profits.

### The influence of the subsidy coefficient on the retail price

**Fig 2:**
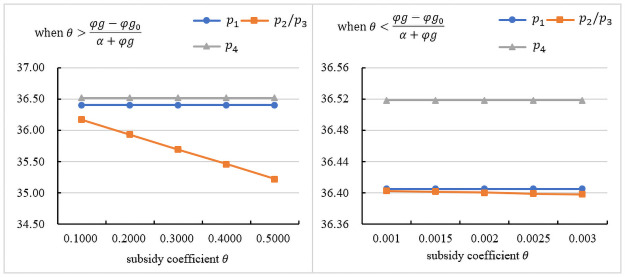
The influence of the subsidy coefficient on the retail price.

Fig 2 indicates the impact of *θ* on the retail price under different subsidy objects. Fig 2(a) presents the trend in the retail price under $\theta >\frac{\varphi g-\varphi g_{0}}{\alpha +\varphi g}$. Fig 2(b) presents the trend in the retail price under $\theta <\frac{\varphi g-\varphi g_{0}}{\alpha +\varphi g}$. As seen in the figures, when the government subsidizes the manufacturer or the assembler, the retail price is the lowest, and it is inversely proportional to the subsidy coefficient. Because the government subsidizes the manufacturer, the wholesale price will be reduced, which can reduce the assembler’s cost, so the retail price will be reduced. As *θ* increases, the retail price decreases more. When the government subsidizes the assembler, the government subsidy can bring additional profits to the assembler. The assembler actively responds to the government subsidy policy and can also obtain more subsidies. According to *D*_1_(*p*, *δ*), a lower retail price can also increase demand. Therefore, the assembler will lower the retail price. When the government subsidizes customers, the retail price is the highest. Because the government subsidizes consumers, increasing the wholesale price and the assembly rate leads to increased costs for the assembler. The assembler aims at maximizing its interest, which will increase the retail price.

### The influence of the subsidy coefficient on the assembly rate

**Fig 3:**
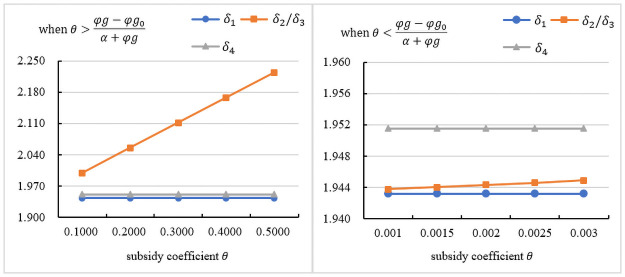
The influence of the subsidy coefficient on the assembly rate.

Fig 3 indicates the impact of *θ* on the assembly rate under different subsidy objects. As seen in the figures, regardless of who the subsidy objects are, government subsidies can increase the assembly rate. Fig 3(a) presents the trend in the assembly rate under $\theta >\frac{\varphi g-\varphi g_{0}}{\alpha +\varphi g}$. As seen in the figures, when the government subsidizes the manufacturer or the assembler, the assembly rate is the highest, and it is proportional to *θ*. When the government subsidizes the assembler, it can bring additional profits to the assembler. When the government subsidizes the manufacturer, it can reduce the assembler’s cost. Therefore, the assembler is willing to increase the assembly rate. Fig 3(b) presents the trend in the assembly rate under $\theta <\frac{\varphi g-\varphi g_{0}}{\alpha +\varphi g}$. As seen in the figures, when the government subsidizes customers, the assembly rate is the highest. In summary, it is better for the government and consumers to subsidize the assembler or the manufacturer. In this case, the retail price is lower, which can attract more consumers, increase demand, and be more conducive to the promotion of prefabricated construction. Although the assembly rate is not optimal, it is also improved compared to that with no subsidy.

### The influence of the subsidy coefficient on the manufacturer’s profit

**Fig 4:**
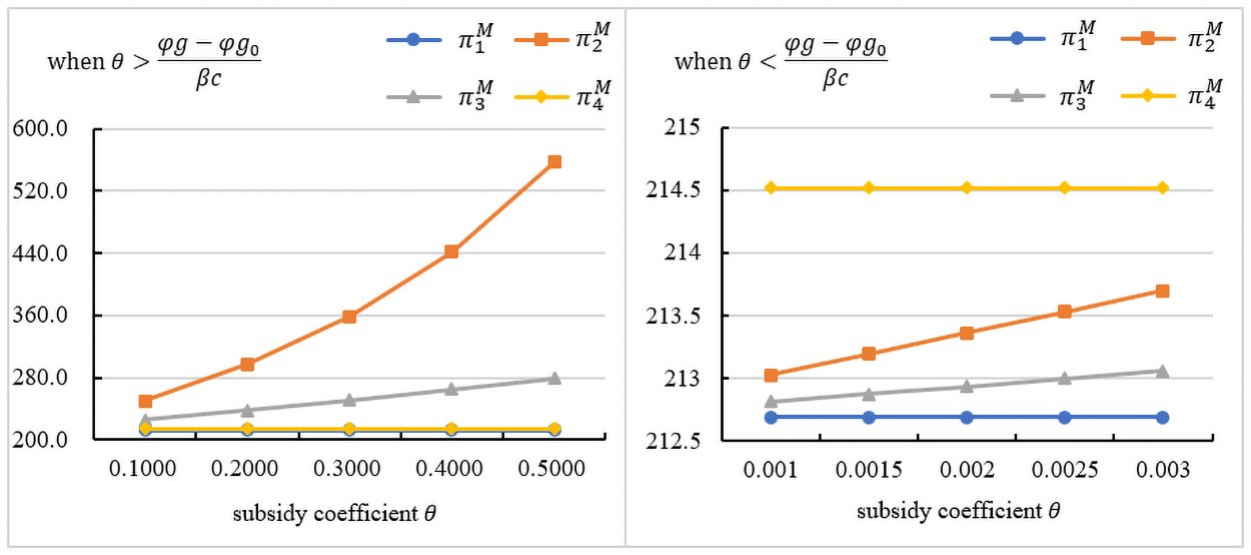
The influence of the subsidy coefficient on the manufacturer’s profit.

Fig 4 indicates the impact of *θ* on the manufacturer’s profit under different subsidy objects. As seen in the figures, regardless of who the subsidy objects are, government subsidies can increase the manufacturer’s profit. Fig 4(a) presents the trend in the manufacturer’s profit under $\theta >\frac{\varphi g-\varphi g_{0}}{\beta c}$. As seen in the figures, when the government subsidizes the assembler, the manufacturer’s profit is the highest, and it is proportional to *θ*. When the government subsidizes the assembler, the manufacturer will increase the wholesale price. In response to the subsidy policy, the assembler will reduce the retail price and increase the assembly rate, which will increase demand. A higher wholesale price and higher demand will bring more profits to the manufacturer. However, when the government subsidizes the manufacturer, the manufacturer will lower the wholesale price. Although the demand increases, it will reduce the manufacturer’s profit. Fig 4(b) presents the trend in the manufacturer’s profit under $\theta <\frac{\varphi g-\varphi g_{0}}{\beta c}$. As seen in the figures, when the government subsidizes consumers, the manufacturer obtains optimal profit. When *θ* decreases, the manufacturer’s profit will also decrease. However subsidizing consumers can increase demand and bring more profits to the manufacturer. In summary, when $\theta >\frac{\varphi g-\varphi g_{0}}{\beta c}$, it is better for the manufacturer to subsidize the assembler. When $\theta <\frac{\varphi g-\varphi g_{0}}{\beta c}$, it is better for the manufacturer to subsidize the customers.

### The influence of the subsidy coefficient on the assembler’s profit

**Fig 5:**
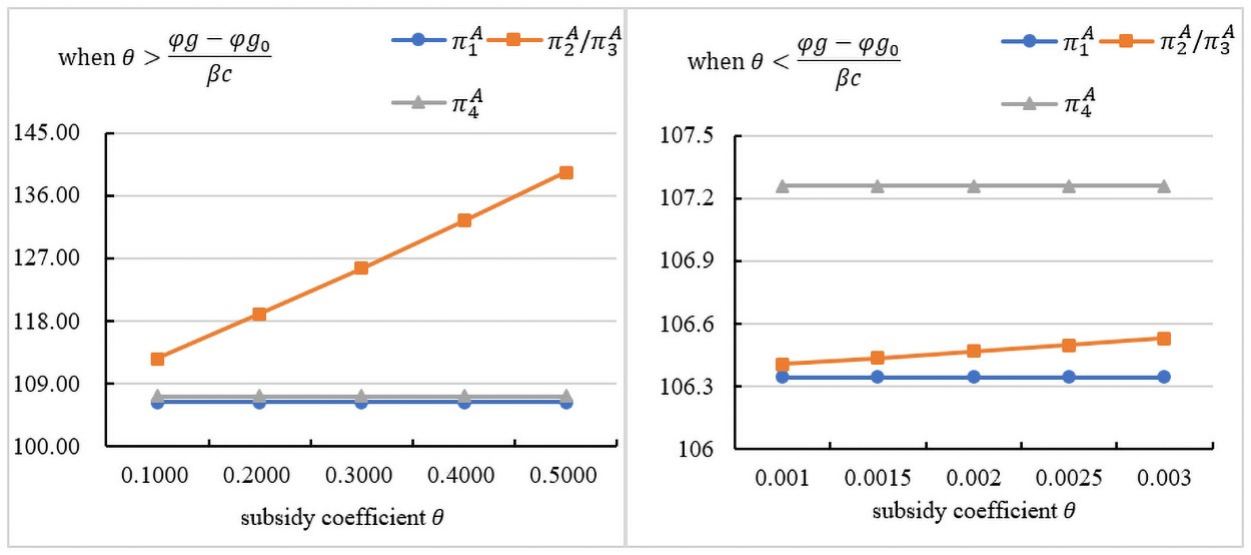
The influence of the subsidy coefficient on the assembler’s profit.

Fig 5 indicates the impact of *θ* on the manufacturer’s profit under different subsidy objects. As seen in the figures, regardless of who the subsidy objects are, government subsidies can increase the assembler’s profit. Fig 5(a) presents the trend in the assembler’s profit under $\theta >\frac{\varphi g-\varphi g_{0}}{\beta c}$. As seen in the figures, when the government subsidizes the assembler or the manufacturer, the assembler obtains optimal profit, and it is proportional to *θ*. When the government subsidizes the manufacturer, the wholesale price will decrease, and *ω* decreases as *θ* increases, which can reduce the assembler’s cost. The assembler reduces the wholesale price and increases the assembly rate, which can also increase the demand and make it more profitable. When the government subsidizes the assembler, it can directly bring additional profits, and it will increase with the increase of *θ*. According to *D*_1_(*p*, *δ*), lowering the wholesale price can increase the demand for prefabricated construction. These will make the assembler more profitable. Fig 5(b) presents the trend in the assembler’s profit under $\theta <\frac{\varphi g-\varphi g_{0}}{\beta c}$. As seen in the figures, when the government subsidizes consumers, the assembler obtains optimal profit. When *θ* decreases, the assembler’s profit will also decrease. However, subsidizing consumers can increase demand and bring more profits to the assembler. In summary, when $\theta >\frac{\varphi g-\varphi g_{0}}{\beta c}$, it is better for the assembler to subsidize the assembler or the manufacturer. When $\theta <\frac{\varphi g-\varphi g_{0}}{\beta c}$, it is better for the assembler to subsidize the customers.

## Conclusions and future research

China is vigorously developing prefabricated construction, but the country’s application of prefabricated construction still lags behind due to its high cost. For this reason, the government has introduced subsidy policies for different objects. However, enterprises’ decision-making environment will be more complicated after considering government subsidies, and subsidy policies for different subsidy objects will have different impacts on the manufacturer’s wholesale price, assembler’s retail price, and assembly rate. Therefore, it is necessary to study the pricing and assembly rate decisions of manufacturer and assembler under government subsidy policies. In this study, a two-echelon prefabricated construction supply chain comprising the manufacturer and the assembler is considered. Then, a profit model for both parties is constructed focusing on a government-subsidized assembler, a government-subsidized manufacturer, and government-subsidized consumers.

Through research, we solved the two issues raised in “Introduction”. The optimal pricing and assembly rate decisions of both parties in the three cases are obtained. The impact of government subsidies on pricing and profit is also examined. We concluded that government subsidy policies can bring more profits to prefabricated construction enterprises; and that enterprises are more willing to adopt prefabricated construction. In addition, doing so it can reduce the retail price, attract more consumers, and increase demand. This is conducive to the promotion of prefabricated construction. Through comparison and numerical analysis, we also find that when the government subsidizes enterprises more, it is better to subsidize the assembler. Consumers can obtain a lower retail price, which can attract more customers and increase demand. Enterprises can obtain more profits and increase the assembly rate, which is beneficial to the promotion of prefabricated construction. When the government subsidizes customers more, it is better for the assembler and the manufacturer to subsidize customers, because they can obtain more profits. It is better for the government and customers to subsidize the assembler or the consumer. The government has to consider whether the retail price of prefabricated construction is acceptable to consumers. In this case, consumers can obtain a lower retail price. Although the assembly rate and enterprises’ profits are not optimal, they have also been improved. In addition, when the government directly subsidizes enterprises, the enterprises will actively cooperate with the subsidy policy and are more willing to adopt prefabricated construction. Doing so will benefit the promotion of prefabricated construction.

This study considers the influence of government subsidies on prefabricated construction supply chains’ pricing and assembly rate decisions and enriches the literature on prefabricated construction supply chains. It would be interesting to extend our study to examine the influence of government subsidies in a prefabricated construction supply chain consisting of multiple manufacturers and an assembler. Moreover, this study only studies the impact of government subsidies on the supply chain’s decisions under decentralized decisions, and does not consider the impact under centralized decisions. Therefore, the authors’ future research direction is to study the influence of government subsidies on supply chain decisions under centralized decisions, compare the findings with those related to decentralized decisions, and design the coordination strategy of the prefabricated construction supply chain.

## Supporting information

S1 File Data file. (XLSX)
